# Using Eye Movement Data Visualization to Enhance Training of Air Traffic Controllers: A Dynamic Network Approach

**DOI:** 10.16910/jemr.11.4.1

**Published:** 2018-08-08

**Authors:** Saptarshi Mandal, Ziho Kang

**Affiliations:** University of Oklahoma, USA

**Keywords:** Eye tracking, Eye movement, Scanpath, Air Traffic Control, Dynamic network, Visualization

## Abstract

The Federal Aviation Administration (FAA) forecasted substantial increase in the US air traffic volume creating a high demand in Air Traffic Control Specialists (ATCSs). Training times and passing rates for ATCSs might be improved if expert ATCSs’ eye movement (EM) characteristics can be utilized to support effective training. However, effective EM visualization is difficult for a dynamic task (e.g. aircraft conflict detection and mitigation) that includes interrogating multi-element targets that are dynamically moving, appearing, disappearing, and overlapping within a display. To address the issues, a dynamic network-based approach is introduced that integrates adapted visualizations (i.e. time-frame networks and normalized dot/bar plots) with measures used in network science (i.e. indegree, closeness, and betweenness) to provide in-depth EM analysis. The proposed approach was applied in an aircraft conflict task using a high-fidelity simulator; employing the use of veteran ATCSs and pseudo pilots. Results show that, ATCSs’ visual attention to multi-element dynamic targets can be effectively interpreted and supported through multiple evidences obtained from the various visualization and associated measures. In addition, we discovered that fewer eye fixation numbers or shorter eye fixation durations on a target may not necessarily indicate the target is less important when analyzing the flow of visual attention within a network. The results show promise in cohesively analyzing and visualizing various eye movement characteristics to better support training.

## 1. Introduction

The Federal Aviation Administration (FAA) forecasted a 1.4% annual increase in the US air traffic volume; from currently 43.2 million aircraft to 60.3 million by 2040.(1) However, the currently available number of expert air traffic control specialists (ATCSs) might not be sufficient to handle the anticipated increase of air traffic volume. Additionally, the current training completion time of the air traffic controllers takes many years of intensive training.(2) Therefore, the FAA has been trying to find ways to efficiently train the FAA Academy candidates.

One of the critical tasks of ATCSs is to detect and mitigate possible aircraft conflicts (i.e. possible collisions) through visually scanning the radar screen. The ATCSs look for conflicting situations between aircraft pairs (or groups) to resolve it and guide them to their destination in a safe/timely manner. Thus, the ATCS’s task involves a significant amount of visual scanning of the radar display and, subsequently, cognitive processing of the observed information to take necessary actions. Eye-mind hypothesis(3) showed there exists high correlation between the eye movement (EM) data and the cognitive process of an observer. Kang and Landry(4) demonstrated that exposing novice controllers to the visual scanpath of the expert ATCSs improved their overall scanning efficiency by reducing their false positive cases of conflict detection among aircraft. On Similar lines, Rudi, Kiefer and Raubal(5) demonstrated that visualization of EM data of pilot’s working in a cockpit might prove useful for flight instruction purposes. Therefore, if we could effectively analyze, visualize, and interpret experts’ eye movement characteristics, we might be able to use those findings to train the candidates or novices.

**Figure 1.**
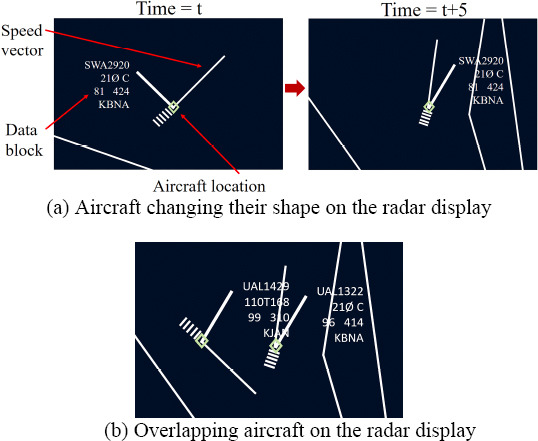
Dynamic aspects of aircraft representation on the radar display of an ATCS: (a) Location of the data blocks changes relative to the aircraft location. (b) Two overlapping aircraft.

Effective analysis of ATCSs’ visual scanning process is challenging. The Radar display has, a large number of dynamic targets (i.e. aircraft on radar display) which have dynamic properties (e.g. radar representation of an aircraft can change their shape and position with time). Figure 1 represents the dynamic aspect of the radar representation of aircraft.

Visualization of ATCSs’ EM data includes two steps. The first step involves developing a time-ordered mapping between the eye fixations (EFs) and the aircraft on the display. The second step consists of characterization of the developed scanpath sequences. To develop the mapping function in case of moving and overlapping targets, Dynamic Areas of Interest (AOIs) can be created which are dynamic convex boundaries that fits the moving targets and considers the visual angle accuracy.(6, 7, 8) However, as the number of targets increases, visualization of the scanpath sequences becomes challenging using the widely used visualization methods such as point based and AOI based methods(9) (explained below in Background section), since these methods might create visual clutter when numerous targets are visualized. In addition, air traffic has a dynamic nature, meaning that an aircraft can dynamically move within the radar display for a certain amount of time, and/or two or more aircraft can overlap on one another. As a result, any aircraft’s relevance from the visual scanning point of view evolves with time. The prevalent visualization methods are unable to handle this dynamic aspect of the ATCS’s visual scanning process.

In addition, other existing pivotal researches(10, 11, 12) focus more on EF numbers and EF durations or simpler forms of scanpaths (explained in Background section); however, for a dynamic task such as air traffic control task, how the multiple targets (e.g. aircraft) are observed as a network (using the various EM transition characteristics) can also be important, meaning that even if there were fewer number of EFs or less durations, a target can be considered important if it plays a crucial role in the EM flow among multiple targets or acts as a bridge between two disconnected groups of targets. Furthermore, if we only consider the number of EFs on the aircraft, it might produce an incorrect interpretation about the important aircraft. For example, consider a case when an aircraft has just entered the radar display. There is high chance that this new entrant aircraft might receive a substantial amount of EF duration, as the ATCSs might want to know about its destination, altitude, and other details; however, this new aircraft might not be important in terms of conflict resolution with the already existing set of aircraft on the display. As a result, the ATCSs will not fixate again on this aircraft, rendering it unimportant in terms of the overall scanning strategy. Zhang, Ren and Wu(13) provided valuable findings using static networks in the air traffic control domain, but the dynamic aspects and the issues raised above were not addressed.

Therefore, we need an improved analysis framework which will help us (1) develop visualization methods which can represent the EM data with a large number of targets with less visual clutter, (2) find measures that can accommodate the dynamic aspects of the moving targets, and (3) integrate the visualizations and measures for effective analyses and interpretations.

In this paper, we provide several approaches to address the issues raised above. First is to adapt the dynamic network (DNet) approach(11) and modify its structural components for visualizing the EM data of the ATCSs. A DNet is a collection of time-ordered static networks. The DNet visualization enables easy handling of a large number of targets, thereby reducing visual clutter. Being a collection of several networks, the DNet can easily represent the evolution of a target’s importance over time and the dynamics of visual scanning characteristics. The adapted DNet is aligned with three “vertex importance measures” such as “indegree,” “closeness,” and “betweenness”(14) to better determine important targets. Furthermore, two types of normalization procedures (i.e. percent normalization and distance normalization) are introduced that calculate the relative amount of visual attention given to a target in comparison to the maximum values obtained for a specific task. Finally, we adapted the dot plots and bar plots to either better represent the evolution of the important targets or compare the vertex importance measures among the participants. Note that we will replace the term “vertex” with “target” or “AOI” for easier understanding.

## 2. Background

### 2.1 Eye movement (EM) visualizations: Point based, area of interest (AOI) based, and hybrid

Blascheck et al(9) have categorized the various EM visualization methods into point based, AOI based, and hybrid visualizations. Summaries and issues are as follows.

Existing point based visualization methods, e.g. timeline visualization,(15) scanpath visualization,(16) attention maps,(17) space-time cubes,(17) represent the time-ordered horizontal and vertical coordinates of the EFs occurring on the display. These methods are effective on visualizing the exact EF locations to unravel important regions (in absence of predefined targets) when given static stimuli. However, due to the visual angle error of the eye trackers, it is challenging to map the EFs with small and dynamic multi-element targets making it difficult to apply the point based methods.(18) In addition, our interest is in investigating which moving targets were focused upon rather than the physically fixed area within a display.

On the other hand, existing AOI based visualization methods allows EM analysis based on either pre-defined region or target on the display. The AOI based methods have been categorized into timeline and relational AOI visualizations.(9) Time­line AOI visualizations such as parallel scanpath,(19) scarf plot,(20) and AOI river plot(21) on developing effective methods to represent the AOIs that have been fixated upon at various time intervals. However, these methods are challenging to apply for long duration tasks having large number of targets (e.g. twenty or more targets) and frequent EF transitions between them (e.g. air traffic control task).

Relational AOI visualization methods are more appropriate to handle the issues raised above through visualizations using circular heat map transition diagram,(10) transition matrix,(12) and network visualization.(11) These methods visualize the aggregated EM data by showing the relationship that exists between he AOIS in terms of the EF transitions between them, unlike the timeline approaches. Relational AOI based approaches do not represent the physical location of the AOIs on the display. In detail, in circular heat map visualization,(10) AOIs are represented as segments of a circular layout (using different colors and sizes) and the EF transitions are shown by directed arrows between the circular segments. Transition matrix visualization(12) represents the EF transitions among the AOIs in a tabular fashion. The most appropriate approach to address the issues of a dynamic task is through the network visualization that shows the AOIs as vertices and the EF transitions between AOIs as the directed edges between the vertices of a network.(11, 18, 22, 23)

However, if we try to apply the relational AOI visualization methods, we often run into possible visual clutter issues if there are large number of targets and it can be difficult to represent all the EM characteristics using the existing network approach. The following subsections 2.2. and 2.3 provide summaries of the DNet mathematical framework and how various EM network characteristics can be integrated based on time intervals.

### 2.2. Mathematical framework of DNet

A DNet is as a sequence of static networks (also called networks), where each constituent network is associated with a time interval.(24) If the total time duration of the collected EM data is divided into T time intervals, then a DNet representing such a data is written as $DynN\;=\{N_{1},N_{2},..N_{\text{t }\;},..,N_{T}\}$*,* where $N_{\text{t }\;}$ is the network for time interval t, where t=1,2,..,T.

A network $N_{t\;}$ is written as $N_{\text{t }\;}=(V_{\text{t }\;},E_{\text{t }\;},M_{\text{t }\;})$*,* where, $V_{\text{t }\;}$ is the set of vertices (AOIs for the present study), $E_{\text{t }\;}$ is the set of edges (EF transitions for the present study) between the vertices, and $M_{\text{t }\;}$ is the adjacency matrix which contains all edge weights (amount of EF transitions between AOI pairs).

The set of vertices is written as $V_{\text{t }\;}=(v_{1\;},v_{2},\ldots ,v_{m_{t}\;})$*,* where $m_{t}$ is the number of vertices for time interval t. A network can either have directed or undirected edges, although for EM visualization we only consider directed edges. The set $E_{\text{t }\;}$ consists of ordered pairs of vertices $\left(v_{i},v_{j}\right)$ showing that there exists a directed edge from the vertex $v_{i}$ towards vertex $v_{j}$. Thus, $E_{\text{t }\;}=\left\{e_{\text{ij}}(t)\right|v_{i},v_{j}\in V_{\text{t }\;},\;i\neq j\}$. Lastly, the adjacency matrix is written as $M_{\text{t }\;}={\left[w_{\text{ij}}(t)\right]}_{m_{\text{t }\;}\times m_{\text{t }\;}}$, where, $w_{\text{ij}}(t)$ is the weight of the edge $e_{\text{ij}}(t)$.(25, 26)

### 2.3 DNet for EM visualization

Beck et al(24) provided an exhaustive list of various DNet visualization approaches representing EM data. Depending on the representation of the time variable, various visualization approaches have been categorized into two groups: Animation, and timeline visualization. Animation visualization refers to representing a DNet as an animated sequence of networks. Timeline visualization refers to representing a DNet as a sequence of networks in a single image showing the complete sequence of interactions between the targets. In the present work, we have applied the node-link based timeline approach for representing the DNet, because this visualization helps to preserve the mental map and reduces the cognitive load of the observer.(27)

As noted by various researchers,(28, 29, 30) preserving the mental map (i.e. the abstract structural information layout about a network’s elements that an analyst develops in their mind as they visually scan the visualization) helps in tracing the change in vertex properties and edge paths across different time intervals. Additionally, the timeline visualization, using the node-link approach, provides an intuitive and efficient framework for analyzing the change of states of multiple vertices over time.(31, 32)

However, the existing DNet approach only uses the number and duration of EFs on the AOI to measures the AOI importance. As noted in the introduction, these two raw measures may lead to misleading results in case of dynamic targets. Therefore, it is required to consider other target importance measures to address the highlighted issue.

The next section discusses the three target importance measures (from the network science domain) that can be adapted for analysing AOI importance for dynamic scenarios.

### 2.4 Target (or AOI) importance measures

The three most popular target importance measures in a given network are indegree, closeness, and betweenness.(14, 25, 33) Mandal et al(18) have shown some possibilities in applying the above-mentioned measures to build a basic foundation for the proposed approach in this article. It is noted that we introduce the “time” element (“t”) within the three vertex importance measures to consider the dynamicity.

Indegree of a vertex is defined as the sum of all incoming weights to it from all other vertices in the network. For the present study, incoming weights can be interpreted as the incoming EF transitions to a given AOI. Thus, indegree for the $j^{\text{th}}$ AOI is given as $I_{j}=\sum_{k=1}^{m}w_{\text{kj}}$,(25) where, $w_{\text{kj}}$ is the number of EF transitions from the $k^{\text{th}}$AOI to the $j^{\text{th}}$AOI, and m is the total number of AOIs on the display.

We should note that the indegree measure, shown above, is static in nature. As a result, we modified it to develop the dynamic analogous, where the indegree for an AOI is defined for each of the time interval considered in the DNet framework. Thus, the modified indegree measure for the $j^{\text{th}}$AOI for time interval t is calculated as:

(i) $$I_{j}(t)=\sum_{\begin{array}{c} k=1\\ k\neq j \end{array}}^{m_{t}}w_{\text{kj}}(t)$$

Where, $w_{\text{kj}}(t)$ is the number of EF transitions coming for the $k^{\text{th}}$AOI to $j^{\text{th}}$AOI and $m_{t}$ is the number of unique AOIs in the AOI fixation sequence for time interval t. Large indegree value suggests higher importance for an AOI, as it received large number of EFs. Thus, indegree can be interpreted as a measure of direct attention received by an AOI.

However, indegree measure only considers the local structure (direct EF transitions) around a vertex but neglects the global structure of the network.(33, 34) This issue is addressed by the two measures named “closeness” and “betweenness.”

Note that the network science (or graph theory) includes the concept of “outdegree” however, the indegree values and outdegree values are always the same within the EM network since there is no possibility of an inward single EM transition dividing into two or more outward transitions.

Before we understand the two measures, we need to define the concept of distance between AOIs in a network visualizing EM data. In the present case, we define the distance from one AOI (e.g. ‘A’) to another AOI (e.g. ‘B’) as the inverse of the number of EF transitions from AOI ***A*** towards ***B***. Thus, a large number of EF transitions between AOIs results in a smaller distance between them in terms of the visual scanning strategy.

The closeness of a vertex measures its distance from all other vertices in the network. Thus, higher closeness value for a given vertex means it is easier to access any part of the network from it. In the present study, higher accessibility of an AOI can be interpreted as a greater association (both direct and indirect) with other AOIs in the network. High closeness value suggests that the AOI lies in the central location in terms of the observer’s visual scanning strategy. The closeness for the $j^{\text{th}}$ AOI is given as $C_{j}=\sum_{k=1}^{m}\left(\frac{1}{{d_{\text{jk}}}^{*}}\right)$,(33) where, ${d_{\text{jk}}}^{*}$ is the minimum distance from the $j^{\text{th}}$AOI to the $k^{\text{th}}$AOI (if multiple paths exist) and m is the total number of AOIs on the display. Like the previous indegree measure case, the static closeness measure is also modified to develop its dynamic analogous. Thus, the modified closeness measure for the $j^{\text{th}}$ AOI for time interval t is defined as follows:

(ii) $$C_{j}(t)=\sum_{\begin{array}{c} k=1\\ k\neq j \end{array}}^{m_{t}}\frac{1}{{d_{\text{jk}}}^{*}(t)}$$

Where, ${d_{\text{jk}}}^{*}(t)$ is the minimum distance from the $j^{\text{th}}$AOI to the $k^{\text{th}}$AOI (if multiple paths exist) and $m_{t}$ is the number of unique AOIs in the AOI fixation sequence for time interval t.

In a dynamic scenario, there are instances where due to visual scanning strategy of the observer, an AOI despite receiving small amount of direct attention (low indegree measure) and being present in a non-central location (low closeness value) can still play a significant role by connecting (acting as a bridge between) two groups of AOIs. Such an AOI plays a crucial role in controlling the flow of attention among the other AOIs on the display, and this aspect is measured through the concept of betweenness explained below.

Betweenness for the $j^{\text{th}}$ AOI is defined as $B_{j}=\sum_{l=1}^{m}\sum_{\begin{array}{c} k=1,\;j\neq l\neq k\;\\ \; \end{array}}^{m}\left(\frac{{\text{SP}}_{\text{kl}}^{j}}{{\text{SP}}_{\text{kl}}}\right)$,(33) where ${\text{SP}}_{\text{kl}}$ represents the total number of shortest paths (if multiple paths exist) from the $k^{\text{th}}$AOI to the $l^{\text{th}}$AOI, and ${\text{SP}}_{\text{kl}}^{j}$ represents the number of such shortest paths that pass through the $j^{\text{th}}$AOI. Thus, the modified betweenness measure for the $j^{\text{th}}$ AOI for time interval t is defined as follows:

(iii) $$B_{j}(t)=\sum_{k=1}^{m_{t}}\sum_{\begin{array}{c} l=1\\ \;j\neq l\neq k \end{array}}^{m_{t}}\frac{{\text{SP}}_{\text{kl}}^{j}(t)}{{\text{SP}}_{\text{kl}}(t)}$$

Where, ${\text{SP}}_{\text{kl}}(t)$ represents the total number of shortest paths (if multiple paths exist) from the $k^{\text{th}}$AOI to the $l^{\text{th}}$AOI, and ${\text{SP}}_{\text{kl}}^{j}(t)$ represents the number of such shortest paths which pass through the $j^{\text{th}}$AOI for the time interval t.

## 3. Proposed approach

**Figure 2.**
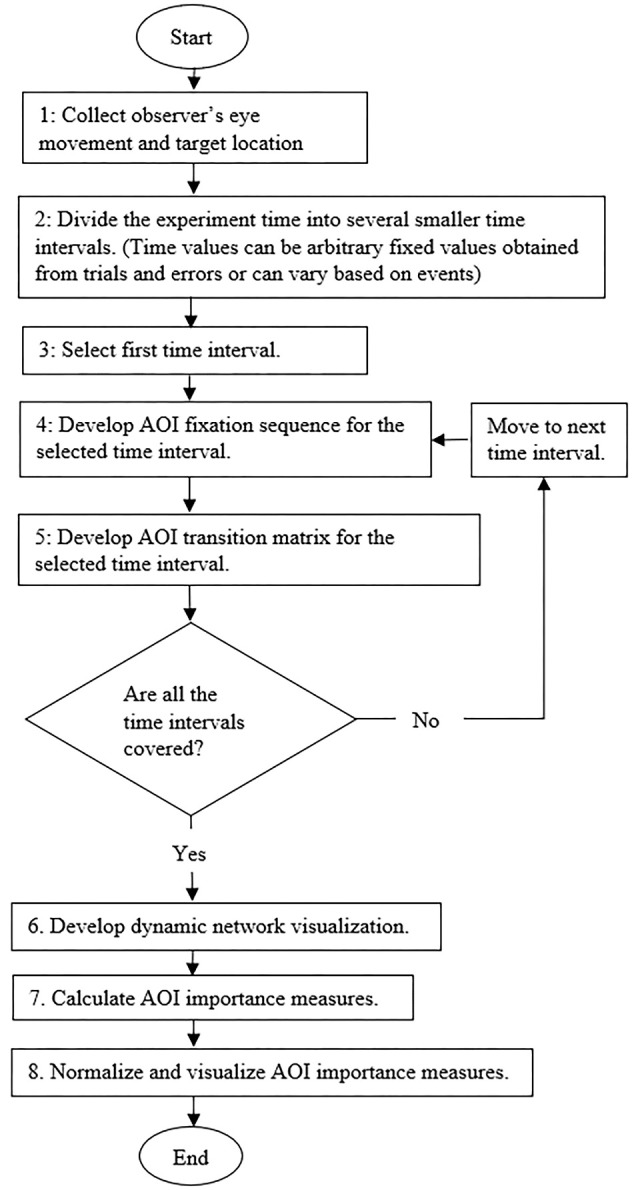
Flowchart showing the various steps of the proposed methodology for analyzing time interval based EM data.

Figure 2 represents the various steps in the proposed methodology for analyzing ATCS’s EM data.

### 3.1 STEP 1. Collect observer’s EM and targets’ location data

The input for the first step is the simulation experimental data. As output for this step, two types of data are obtained: (a) EM data, that consists of horizontal and vertical coordinates of the EF and its associated fixation duration, and (b) target location data, that consists of the pixel coordinates of the various targets on the display.

### STEP 2. Divide the experiment time into several smaller time intervals

The input for step 2 is the time duration (in minutes or hours) for which the experiment has been conducted, and the output are several equal (unequal) sized time intervals which sum to the experimental time duration. For example, consider that the total experiment duration is divided into four equal time intervals (i.e. T=4, see section 2.2). Note that the time intervals can be chosen based on the task characteristics or the researcher’s judgment (i.e. fixed or event-based time intervals).

### 3.3. STEP 3. Select first time interval

The inputs for this step are the various time intervals obtained in the previous step 2. The intervals are indexed and arranged in a time-order sequence. In this step, we start with the the first time interval to initiate developing the AOI fixation sequence.

### 3.4 STEP 4. Develop AOI fixation sequence (i.e. scanpath sequence) for the selected time interval

As input, step 4 receives three things, the time interval selected in step 3, and the EM and target location data for this time interval. As output from this step, we obtain the AOI fixation sequence for the time interval considered. The size of the AOI fixation sequence is directly proportional to the number of EFs in the time interval. The AOI fixation sequence is created for the selected time interval by adapting the approach suggested by Kang et al(7). Creating the AOI fixation sequence involves mapping the EFs with the AOIs. Only those EFs falling within any of the AOI boundaries are considered for AOI fixation sequence development, else they are ignored. AOIs are coded by assigning uppercase letters followed by lowercase alphabets (i.e. A, B, …, a, b). The developed AOI fixation sequence is the collapsed form of a raw AOI fixation sequence. In the collapsed form of the sequence, multiple consecutive fixations of the same AOI is collapsed to a single fixation case (e.g. ***AAA*** is collapsed into ***A***). Thus, a raw AOI fixation sequence ***AABCC*** is collapsed to ***ABC***. In addition, an overlapping AOI case is shown in parenthesis with individual constituent AOIs separated by a semi-colon. For example, if an EF falls within the overlapped region of AOIs ***A*** and ***B***, it is represented as ***(A; B)***. After mapping all the EFs to the AOIs, we obtain a time-ordered sequence of AOIs, that shows which AOIs were fixated and in which order. Table 1 shows a sample AOI fixation sequence for four time intervals in a hypothetical scenario with seven AOIs.

### 3.5 STEP 5. Develop AOI transition matrix for the selected time interval

Step 5 receives the AOI fixation sequence developed in step 4 as input. Afterwards, the AOI fixation sequence is transformed, as per the approach suggested by Noton and Stark,(35) for developing the associated AOI transition matrix, which is the output of this step. The size of the transition matrix depends on the number of unique AOI states (single and overlapped both) in the fixation sequence. The AOI transition matrix shows, in a tabular manner, how many transitions have occurred between various AOIs pairs. For example, Table 2 represents the AOI transition matrix associated with the AOI fixation sequence for time interval 1 in Table 1. The sequence shows there are three EF transitions from AOI ***A*** to ***B*** highlighted in grey within Table 2. A different AOI transition matrix is required for each time interval; thus, before moving to the next step, we need to create the associated AOI transition matrix for each time interval.

**Table 1.** Samples AOI fixation sequences having overlaps

**Time interval**	**AOI fixation sequence**
1	ABABABEBAEAEACACAEA
2	EAEAEAEABABCBC(D; G)C(D;G)
3	C(D;G)C(D;G)C(D;G)CEAEAEFEF
4	EFEFEFCFC(D;G)C(D;G)FC(D;G)

**Table 2:** AOI transition matrix developed from the AOI fixation sequence for time interval 1 within Table 1

**From AOI**	**To AOI**			
	**A**	**B**	**C**	**E**
**A**	0	3	2	3
**B**	3	0	0	1
**C**	2	0	0	0
**E**	3	1	0	0

### 3.6 STEP 6. Develop DNet visualization

The inputs for step 6 are the AOI transition matrix for all the time intervals obtained in step 2. As output from this step, we obtain the DNet representation of the EM data. Developing the DNet involves two steps. First, developing a static network for each of the time intervals considered in STEP 2. Second, arranging the static networks in a time-based order to visualize the DNet. The details of both the steps are given below.

#### 3.6.1 Develop static network for each time interval

The static network is developed by adopting the design principles suggested by Mandal et al(18). Thus, a network’s vertex size is drawn proportional to the number of EFs received by the corresponding AOI and the vertex’s color is based on a sequential multihued color scale, where red color means high EF duration and yellow color means low EF duration occurring on the associated AOI. The thickness of an edge between a vertex pair is proportional to the number of EF transitions occurring between the vertices in the edge’s direction. Therefore, for the example shown in Table 1, we have four time intervals, thus the DNet is written as $\text{DynN}\;=\{N_{1},N_{2},N_{3\;},N_{4}\}$, where $N_{i}$ is the static network for time interval i(i=1,…,4).

#### 3.6.2 Visualize the DNet

Before visualizing the DNet, the vertices of the component static networks are to be arranged in a specific layout for mental map preservation. We have used the rectangular grid layout for this purpose; in which, we start from the left bottom corner and move towards the right, ending at the top right corner. We sort the AOI positions using the natural ordering of English letters and putting uppercase AOI groups first and then lowercase AOI groups. The single AOIs are followed by overlapped AOI cases in the grid layout. The overlapped AOI cases are arranged in an increasing order of constituent AOI numbers, i.e. an overlapped AOI case with two AOIs comes before a case with three AOIs. For example, overlapped AOI ***(A;B)*** comes before ***(A;B;C)***, which comes before ***(A;B;C;D)***, and so on.

**Figure 3.**
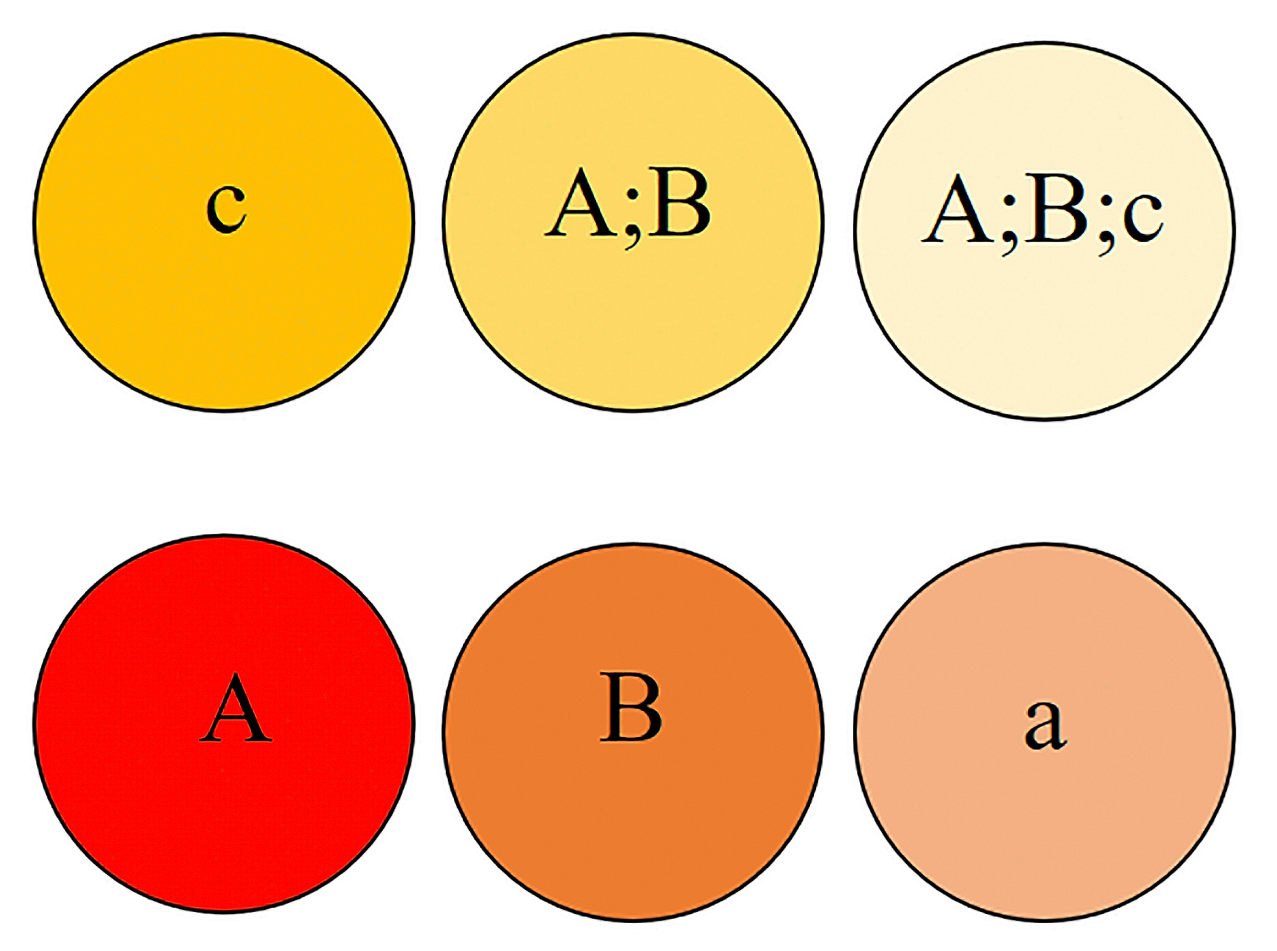
Sample AOI ordering scheme for the grid layout used in designing the networks in the DNet framework.

Consider an AOI fixation sequence that has n unique AOI states (including both single and overlapped AOIs). Therefore, the number of columns (number of AOIs in a single row) in the grid layout will be the smallest integer greater equal to $\sqrt{n}$. Figure 3 represents a sample ordering scheme of six AOIs in a rectangular grid layout.

**Figure 4.**
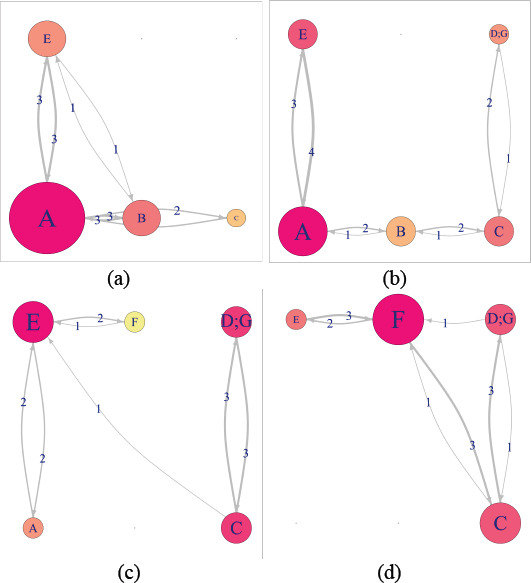
Sample DNet visualization of EM data for the hypothetical scenario described in section 3.4. The DNet consists of four time intervals. The figure shows the important AOIs for each time interval, and how the importance of various AOI’s is changing with time. Sequentially: (a) Time interval 1. (b) Time interval 2. (c) Time interval 3. (d) Time interval 4.

Once the component static networks are arranged in a grid layout, we visualize the DNet by arranging the static networks in a time-ordered sequence. Figure 4 represents a sample DNet visualization of the EM data for a hypothetical scenario (see section 3.4). Note that the relative location of all AOIs in Figure 4 remains constant in each of the networks corresponding to various time intervals. For example, AOI ***A*** is placed at the bottom row first column in all the constituent networks. This constant relative position of each AOI helps in their navigation across various time intervals and thus helps to preserve the mental map of the observer. The DNet has four time intervals, and the AOI fixation sequences for all these intervals is shown in Table 1. We should note that, due to the dynamic nature of the AOIs and typical scanning strategy of ATCSs, some AOIs may not be fixated upon despite being present on the display. In addition, the overlapped AOI cases arise in the AOI fixation sequence only if they are fixated upon by the ATCSs. Theoretically, there can be many possible overlapped AOI cases as compared to what is observed in real life experimental data. For example, with n unique AOIs, theoretically, we can have $\sum_{r=1}^{n}\left(\frac{n}{r}\right)=\sum_{r=1}^{n}\frac{n!}{r!\left(n-r\right)!}$ possible AOIs cases (including both single and overlapped cases) on the display. Although, not all overlapped AOI states will appear on the display and even if they occur not all of them will be fixated upon by the ATCSs. This is also the case with single AOI cases.

Thus, it is computationally expensive and inefficient approach to consider all those AOI states which have not being fixated at all and thus does not appear in the AOI fixation sequence. Consequently, we only consider those AOI states for visualization and analysis which appear at least once in the AOI fixation sequence of the ATCS’s. As a result, we ignore those AOIs which, despite being present on the display, received no EFs from the ATCSs. Therefore, not all AOIs appear in the DNet visualization for each of the ATCSs, which results in a change in the relative position of each AOI in the grid structure of the DNet visualization. In addition, for comparing various visual scanning strategies, we only consider the AOI cases which are common to all the AOI fixation sequences of various ATCS’s.

### 3.7 STEP 7. Calculate target (or AOI) importance measures

Step 7 involves calculating the measures using equations (i), (ii), and (iii). As a result, we provide the DNet as the input to this step, and as output we obtain all three importance measure values for each of the AOIs with respect to all the time intervals considered in the DNet. For example, consider the DNet visualization in Figure 4, where the indegree value of AOI ***B*** changes from 4 to 3 as we move from the time interval 1 to 2 (Figure 4 (a)-(b)). For the given DNet in Figure 4, to calculate the closeness and betweenness value for AOI ***B***, we first demonstrate how to calculate the distance between the AOIs. For example, in the first time interval, the distance from AOI ***B*** to ***A*** is given by $d_{\text{BA}}\left(t\right)=\frac{1}{w_{\text{BA}}\left(t\right)}$ and substituting t=1, we get${\;\text{ d}}_{\text{BA}}\left(1\right)=\frac{1}{3}$.

To calculate the minimum distance between two AOIs consider Figure 4 (a), where there are two EF transition paths from AOI ***B*** to ***E***: Direct path from AOI ***B*** to ***E***, which has edge weight 1; indirect path from AOI ***B*** to ***A*** and then to ***E***, which has edge weights 3 and 3, respectively. Thus, the shortest distance from AOI ***B*** to ***E*** is defined as the path having the minimum distance of ${d_{\text{BE}}}^{*}\left(t\right)=\min\left[d_{\text{BE}}\left(t\right),\;\left(d_{\text{BA}}\left(t\right)+d_{\text{AE}}\left(t\right)\right)\right]$. For the first time interval (t=1), we obtain ${d_{\text{BE}}}^{*}\left(1\right)=\min\left[1/1,\;\left(1/3+1/3\right)\right]\;=\min\left[1,\;2/3\right]=2/3$. Thus, for the first time interval, the closeness and betweenness value of AOI ***B*** is given by 2.375 and 8, respectively.

### 3.8 STEP 8. Normalize and visualize target (or AOI) importance measures

The last step involves normalizing the calculated measures and subsequently visualizing the normalized measure values. Thus, the obtained AOI importance measures in step 7 acts as input for this step, and as output we obtain the normalized measure values accompanied by their visualization. Equation (i), (ii), and (iii) shows that the three measures both depend on the number of AOIs and the amount of EFs. In addition, these measures also have different units, as a result, they are incommensurable. For the present dynamic scenario, both the number of AOIs and EFs are not constant for the different time intervals considered in the DNet analysis. In addition, to compare the AOI importance values across various time intervals and across multiple observers, we need to eliminate the units of importance measures, thereby bringing them to a similar scale. To address this issue, we present two normalization options.

#### 3.8.1 Percent normalization

Percent normalization refers to dividing an importance measurement of an AOI by the sum of the same measurement of all the AOIs. The percent normalized indegree value of an AOI shows the percentage share of the total number of EFs received by an AOI. Thus, it can be interpreted as the percentage of the total attention attributed to an AOI. The percent normalized indegree value of the $j^{\text{th}}$AOI for time interval t is calculated as:

(iv) $$\bar{I_{j}}(t)=\frac{{\;\text{ I}}_{j}(t)}{\sum_{j=1\;}^{m_{t}}{\;\text{ I}}_{j}(t)}$$

Where, $\bar{I_{j}}(t)$ is the percent normalized indegree value for time interval t. We get, $0\leq \bar{I_{j}}(t)\leq 1$ and $\sum_{j=1\;}^{m_{t}}\bar{I_{j}}\left(t\right)=1$.

#### 3.8.2 Distance normalization

Distance normalization refers to calculating how far away a given importance measurement of an AOI is from the maximum value observed for that measurement within a time interval. The distance normalization process is defined for all the three target importance measures. The distance normalized measure value of an AOI can be interpreted as the relative amount of attention given to an AOI as compared to the maximum amount of attention given to any AOI. The distance normalized measure for the $j^{\text{th}}$AOI, for time interval t, is calculated as:

(v) $$\tilde{{⌀}_{j}}(t)=\frac{{\;⌀}_{j}\left(t\right)-\min_{j}{\;⌀}_{j}\left(t\right)}{\max_{j}{\;⌀}_{j}\left(t\right)-\min_{j}{\;⌀}_{j}\left(t\right)}$$

Where, $\max_{j}{\;⌀}_{j}\left(t\right)$, $\min_{j}{\;⌀}_{j}\left(t\right)$ and $\tilde{{⌀}_{j}}(t)$ is the maximum, minimum, and distance normalized value of the measure ${\;⌀}_{j}(t)$ respectively ($0\leq \tilde{{⌀}_{j}}(t)\leq 1$).

${\;⌀}_{j}(t)$ is applicable for representing any of the three target importance measures (i.e. indegree, closeness, and betweenness) whereas percent normalization is useful for only indegree due to manner in which the measures are calculated (see sections 2.4 and 3.7).

#### 3.8.3 Target (or AOI) importance measure visualization

Once the values of the target importance measures are normalized, the last step involves their subsequent visualization. We provide a couple of examples of visualization approaches: Dot plot based on multiple time intervals (for a single observer and single vertex importance measure) and bar plot based on multiple participants (for a single time interval and single vertex importance measure). There can be various ways to visually represent the combinations of importance measures, normalization methods, multiple time intervals, and number of participants. Another example of a bar plot that compares all importance measures of a single participant for a single time frame is also provided in the Results section.

##### 3.8.3.1 Adapted dot plot visualization

**Figure 5.**
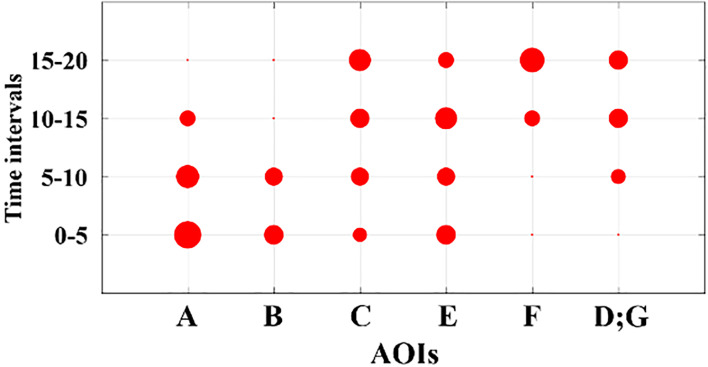
Sample dot plot showing the evolutions of the indegree measurements (for both single and overlapping AOIs) based on time frames using the results shown in Figure 4. Note that the time intervals need not be fixed but can be determined differently (e.g. dividing the time intervals based on events).

Dot plots can better represent the evolution of the visual attention across various time intervals. We have adapted the dot plot to visualize the percent normalized indegree measure for all the time intervals considered in the DNet framework. Using the normalized measurements helps in comparing the relative significance of AOIs within a single time interval and how an AOI’s importance is changing across various time intervals. Figure 5 shows a sample dot plot for the normalized indegree measure for all the AOIs presented in the DNet in Figure 4. In Figure 5, the size of each dot is proportional to the percent normalized indegree value of the AOI for the given time interval.

##### 3.8.3 Target (or AOI) importance measure visualization

**Figure 6.**
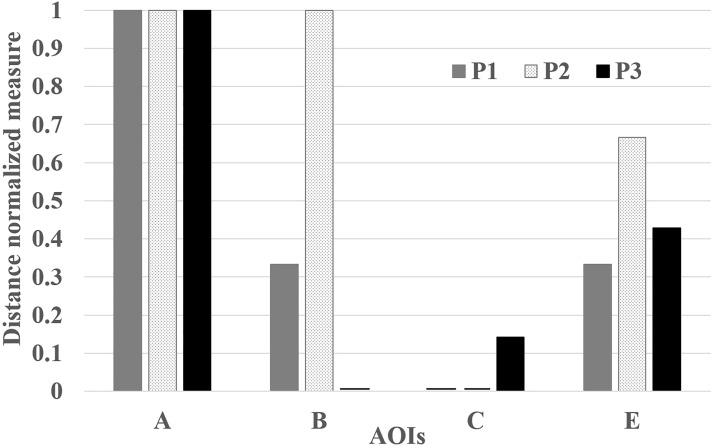
Sample bar plot showing the relative indegree values of various AOIs among multiple observers (i.e. P1, P2, and P3) using the results shown in Figure 4. For this example, the vertical axis represents the distance normalized indegree measure value. Only considering the indegree measure (in this example), AOI A is considered as the important AOI by all participants, whereas AOI B is considered important by only P2.

Bar plots can better assist in comparing a single importance measure among multiple participants or multiple importance measures for a single participant. Figure 6 shows an example of the adapted bar plot that compares the indegree measurements among multiple participants through the distance normalization approach.

## 4. Experiment

The proposed approach was implemented into an air traffic control task. The task involved expert ATCSs observing the radar display to detect possible aircraft collisions. Details are as follows.

## 4.1. Participants

Three retired ATCSs (P1, P2 and P3) with over thirty years of experience were participants for the experiment. The ATCSs were recruited with the help of FAA Civil Aerospace Medical Institute (CAMI). Also, three FAA CAMI employees were also involved in the experiment as pseudo-pilots. They made the necessary maneuverings by following the ATCS’s voice commands. A simulated radio connection was used as the communication channel between the pseudo-pilots and ATCSs.

## 4.2. Apparatus

**Hardware**: A 19.83 × 19.83-inch monitor, with a 2048 × 2048-pixel active display area, was used for displaying the simulated air traffic scenarios. Apart from this, an additional monitor (kept to the right of the simulation monitor) was used to display textual information about the aircraft, e.g. trajectory and possible future conflicts it might encounter (also known as the Enroute Automation Modernization tool). The ATCS’s used a keyboard, placed beneath the simulation display, to provide necessary input commands. FaceLab 5 eye tracker with an accuracy in the range of 0.5−1.0 degree visual angle error and sampling rate of 60 Hz(36) was used to collect ATCSs’ EM data. A threshold of 100 milliseconds was used for defining fixations. The participants were seated within a range of 50-70 cm from the simulated radar display. **Software**: A customized ISim software used by FAA CAMI was used for simulating the enroute air traffic scenario (with an update rate of 1 sec). EyeWorks software was used to process the raw eye tracking data collected through FaceLabs eye trackers.

## 4.3. Scenario and task

### 4.3.1 Scenario

**Figure 7.**
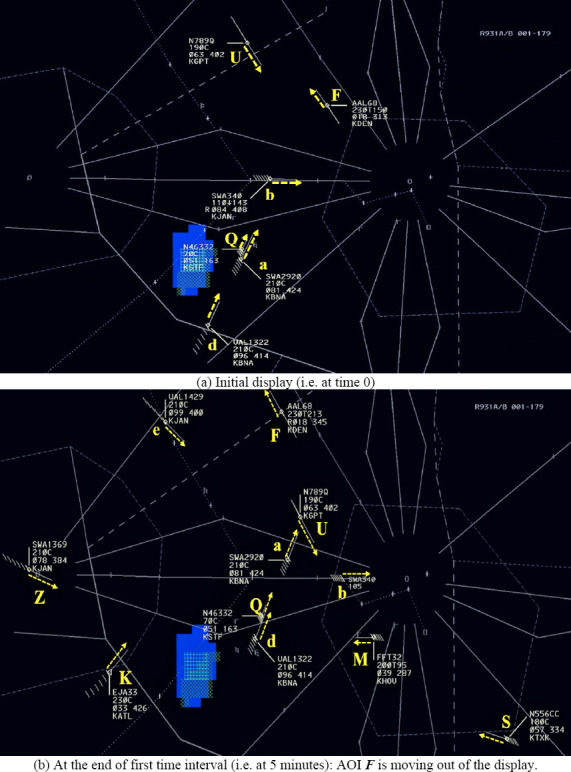
Screen captures at the 0 and 5 minutes. The blue patch shows a weather feature (e.g. thunderstorm) which the aircraft needs to avoid. The characters and arrows in yellow color represent an AOI and the direction of the aircraft, respectively. These characters and arrows were not present during the experiment. Each AOI consists of an aircraft shown as a diamond shape, its direction shown as a vector line, and its associated data block (first line shows the aircraft ID, second line shows its altitude, third line shows its computer ID and speed, and forth line shows its destination). If the altitude changes, the aimed altitude is shown followed by letter “T” and the current altitude. For example, AOI ***F*** is AAL68 (i.e. American Airlines 68) and flying toward northwest. Its current altitude is 21,300 ft (at 5 minutes) and target altitude is 23,000 ft. Its speed is 345 knots (at 5 minutes) and destination is KDEN (i.e. Denver international airport).

For the experiment we used one 20 minutes long simulated enroute air traffic scenario, which was developed and provided by the FAA. This scenario had a total of thirty nine unique aircraft (named as A, B, …, Z, a, b, …, m) with an average of twenty aircraft per frame. The scenario had a minimum of seven and maximum of thirty aircraft per frame. Figure 7 represents a screen capture of the simulated scenario. The scenario update rate was 1 second. The display shows various aircraft and a weather patch in blue color. The aircraft representation shows the direction in which the aircraft is moving (shown by the white line) and data block which contains information about the aircraft’s computer code name, altitude, current speed, and destination airport. For example, in Figure 7, the aircraft N7890 (AOI ***U***) is flying at a constant altitude of 19,000 ft (as shown by 190C) with a speed of 402 knots and it going to KGPT airport (i.e. Gulfport–Biloxi International Airport in Mississippi).

### 4.3.2 Task

The task involved controlling the simulated low altitude enroute airspace using the ERAM system. The ATCSs are required to fulfil two objectives: (1) Route the aircraft through the sector within the display and (2) avoid any conflict scenario by preventing loss of separation (vertical and horizontal separation of 1000 ft and 5 knots respectively) between aircraft. To achieve these objectives, the ATCSs gave voice commands (e.g. change is altitudes, speed and direction of the aircraft) to the pseudo pilots, using the simulated radio, for necessary maneuvering of the aircraft.

## 4.4. Data analysis

For the DNet analysis, the 20 minutes simulated scenario was divided manually, on trial and error basis, into four equal time intervals of 5 minutes. This choice was motivated by the fact that we observed on average an aircraft spends around 5 minutes on the radar display. Event based intervals were not applied in this research since many aircraft appeared and disappeared throughout.

Matlab was used to develop the AOI fixation sequence for each of the four time intervals mentioned above. In addition, igraph package(37) in R software was used to create the dynamic network representation of the AOI fixation sequences and also for AOI importance metric calculation and visualization.

Figure 2 shows there are eight major steps in the proposed methodology. The time complexity (TC) of steps 1-4 for one time interval can be written as ${\text{TC}}_{1-4}=\alpha_{1}e_{t}n_{t}$ , where, $e_{t}$ and $n_{t}$ are the number of eye fixations and number of AOIs in time interval t respectively, and $\alpha_{1}$ is some positive constant. Due to human physiological limitations the value of $e_{t}$ cannot increase arbitrability. Therefore, the governing factor of the time complexity for steps 1-4 is the number of AOIs. Thus, we can approximately write ${\text{TC}}_{1-4}=O(n_{t})$. Taking the worst case scenario, we can find an upper bound for this value by taking the maximum number of AOIs across all time intervals. Let, $n_{\max}$ be the maximum number of AOIs present during any of the time intervals. Thus, we get ${\text{TC}}_{1-4}=\alpha_{1}n_{\max}$. Similarly, the time complexity of step 5 can be written as ${\text{TC}}_{5}=\alpha_{2}{n_{\max}}^{2}$, where $\alpha_{2}$ is some positive constant. For T time intervals the time complexity of step 1-4 and 5 can be written as ${\text{TC}}_{1-4}={\text{Tα}}_{1}n_{\max}$ and ${\text{TC}}_{5}={\text{Tα}}_{2}{n_{\max}}^{2}$ respectively.

As mentioned in section 3.6.2, in a given DNet visualization, the relative location of each AOI remains constant within the various constituent networks for various time intervals. As a result, for drawing the DNet, we need to consider all the unique AOI states that arise in the AOI fixation sequence of an ATCS. Therefore, the time complexity for step 6 can be written as, ${\text{TC}}_{6}={\text{Tα}}_{3}n_{\text{uniq}}$, where, $n_{\text{uniq}}$ is the number of unique AOI states (both single and overlapped) in the AOI fixation sequence and $\alpha_{3}$ is some positive constant. Step 7 consists of calculating three AOI importance measure. The time complexity for indegree, closeness and betweenness metric are ${\text{Tα}}_{4}n_{\max}$, ${\text{Tα}}_{5}({n_{\max}}^{2}-n_{\max})$, ${\text{Tα}}_{6}n_{\max}(n_{\max}-1)(n_{\max}-2)$ respectively. Thus, the time complexity for step 7 can be written as ${\text{TC}}_{7}={\text{Tα}}_{4}n_{\max}+{\text{Tα}}_{5}\left({n_{\max}}^{2}-n_{\max}\right)+{\text{Tα}}_{6}n_{\max}(n_{\max}-1)(n_{\max}-2)$. The time complexity of Step 8 can be written as ${\text{TC}}_{8}={\text{Tα}}_{7}n_{\max}$, where, $\alpha_{7}$ is some positive constant. Adding all the time complexity for step 1-8, the total time complexity for the overall process can be written as: ${\text{TC}}_{\text{tot}}=T\left[\alpha_{1}n_{\max}+\alpha_{2}{n_{\max}}^{2}+\alpha_{3}n_{\text{uniq}}+\alpha_{4}n_{\max}+{\;\text{ α}}_{5}\left({n_{\max}}^{2}-{\;\text{ n}}_{\max}\right)+\alpha_{6}n_{\max}\left(n_{\max}-1\right)\left(n_{\max}-2\right)+\;\alpha_{7}n_{\max}\;\right]$.

On simple algebraic reorganization, we get that: ${\;\text{ TC}}_{\text{tot}}={T[\alpha }_{6}{n_{\max}}^{3}+{n_{\max}}^{2}\left(\alpha_{2}+\alpha_{5}-3\alpha_{6}\right)+\;\;\;\;\;\;\;\;\;\;\;\;\;\;\;\;\;\;\;\;\;\;n_{\max}\left(\alpha_{1}+\alpha_{4}+2\alpha_{6}{+\alpha }_{7}-\alpha_{5}\right)+\alpha_{3}n_{\text{uniq}}]$.

Thus, neglecting the lower order terms of $n_{\max}$, the approx. time complexity of the overall data analysis process reduces to the order of $O({{\text{Tn}}_{\max}}^{3}+Tn_{\text{uniq}})$. Thus, we can see that the number of time interval, maximum number of AOIs within any time interval and the number of unique AOI states in the AOI fixation sequence do impact the processing time of the proposed approach.

## 5. Results

### 5.1. Dynamic graph visualization

**Figure 8.**
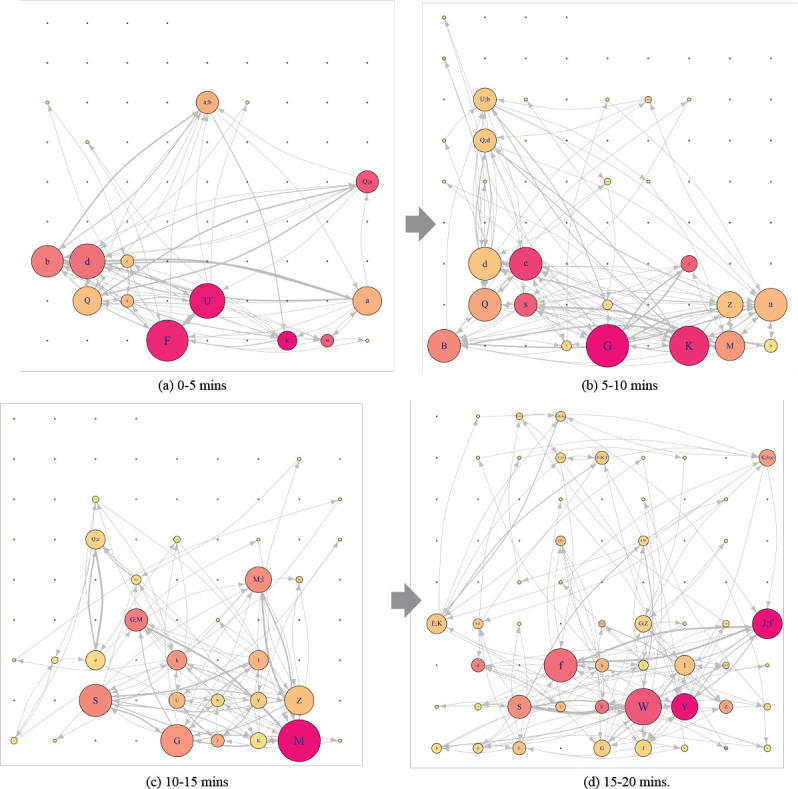
DNet visualisation of the EM data of one ATCS for the simulated enroute air traffic scenario. The figure shows the important AOIs in terms of EF numbers and EF duration for all the four time intervals. It also shows how the importance of various AOIs changes as we move across various time intervals. The relative location of each of the AOIs remains constant across all the static networks associated with different time intervals. For example, AOI ***K*** is placed at the bottom row sixth column for all the constituent static networks.

Figure 8 represents the DNet visualization of the EM data of one ATCS for the simulated scenario shown in Figure 7. In Figure 8 (a) (i.e. time interval 0-5 minutes), AOI ***F*** (i.e. AAL68) and ***U*** (i.e. N7890) are the most important AOIs as they both have most EF numbers (circle size) and longest EF duration (circle color). In addition, there are highest EF transitions between AOI ***F*** and AOI ***U*** (based on the thickness of the link). AOI ***K*** (i.e. EJA33), despite having a small number of EFs, has substantially longer EF duration. AOI ***b*** and AOI ***d*** can also be considered as important AOIs based on how a researcher wants to set the threshold.

For the second time interval (i.e. 5-10 minutes) shown in Figure 8 (b), the important AOIs have changed to AOI

***G*** (newly appeared aircraft not shown in Figure 8(a) and AOI ***K***. Notice that AOI ***F*** has moved out of the display (see Figure 7(b)) and AOI ***U*** is still within the display but AOI ***U*** is not visually attended any longer.

In addition, notice that AOI ***d*** (i.e. UAL1322) has been receiving consistent visual attention throughout the two time intervals 0-5 minutes and 5-10 minutes. Similarly, the important AOIs change for the next two time intervals. In the last time interval, the overlapping AOI (i.e. AOI ***(J;f)***) receives much visual attention. As we move across time intervals from 1 to 4, a visible trend is the increase in the complexity of the network, with increase in the number of AOIs and EF transitions among them.

### 5.2. Adapted dot plot

**Figure 9.**
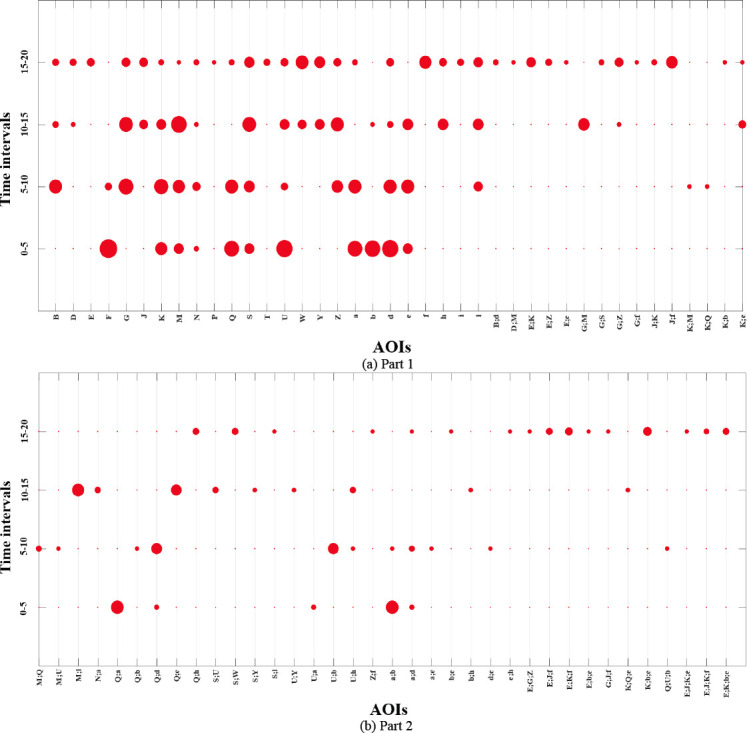
Adapted dot plot visualization of the percent normalized indegree measure value for all AOIs (single and overlapped) present in the DNet visualization in Figure 8.

Figure 9 represents the dotplot visualization of the normalized indegree measure for all the AOIs present in DNet shown in Figure 8. For example, in Figure 9 (a), AOI ***F*** and ***U*** has high importance in first time interval (i.e. 0-5), although, their importance reduces drastically in the subsequent intervals. AOI ***d*** receives consistent visual attention throughout the first two intervals. The indegree results are in accordance with the DNet results in Figure 8 since indegree measures the number of EFs received by an AOI.

The adapted dot plot better shows the evolution of important AOIs. Considering AOI ***K***, we see that its importance initially grows as we move from the first time interval to the second, where it reaches its maximum and then starts decreasing for the last two time intervals. Another noticeable fact is that majority of the overlapped AOI cases have significant importance only in one time interval. These trends took more time to identify when observing the DNet.

### 5.3. Adapted bar plot

**Figure 10.**
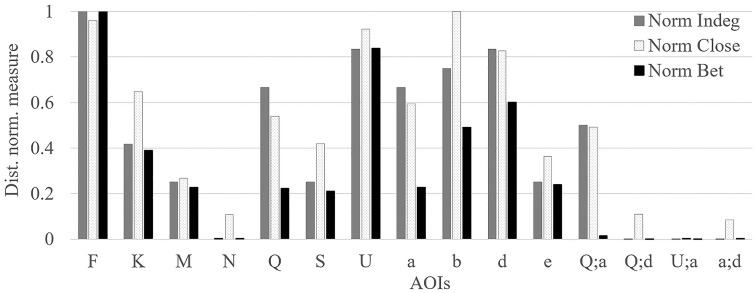
Distance normalized measure value for all AOIs present in the DNet for time interval 0-5 mins for P1 (see Figure 8(a)). “Norm Indeg,” “Norm Close,” and “Norm Bet” refers to normalized indegree, closeness, and betweenness values, respectively. The vertical axis represents the distanced normalized measure value. The vertical axis ranges from 0 to 1, thus it helps to analyze the relative importance of all three measures at a given time interval.

Figure 10 represents the relative importance of various AOIs present in the first time interval of participant 1 (i.e. P1). Again, AOI ***F*** and ***U*** are important, but also, we can identify that AOI ***b*** (i.e. SWA340) and AOI ***d*** can also be important AOIs when we additionally consider the closeness and betweenness values. We can also observe that AOI ***K*** had small EF numbers during the first interval, however, it can be considered as an important AOI due to the long EF duration and relatively high closeness value. Furthermore, we can see that the AOI ***Q*** and AOI ***a*** might be an important AOI considering that AOI ***(Q;a)*** also has moderate indegree and closeness values.

**Figure 11.**
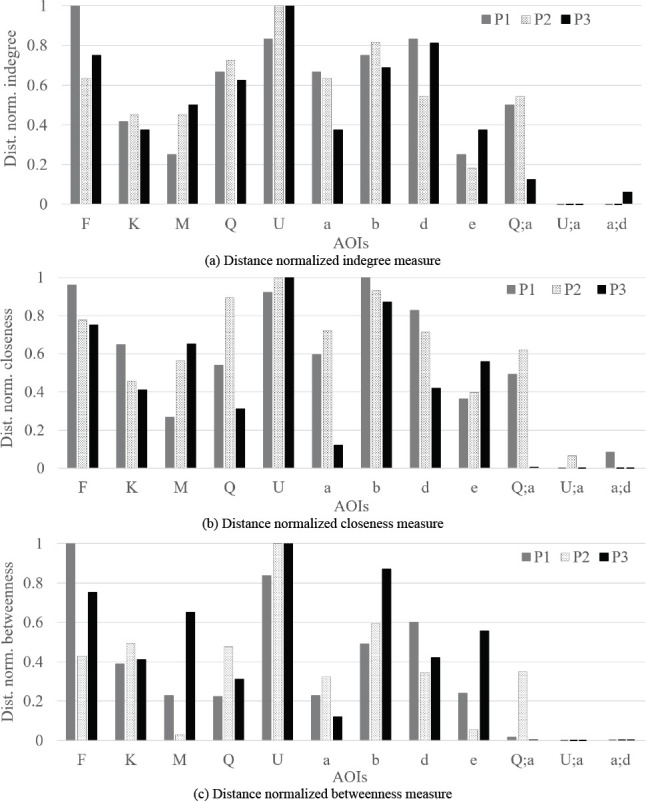
Comparison of three ATCS’s visual scanning strategy by analyzing the distance normalized importance measure values of AOIs. The Figures (a), (b), and (c) shows the relative importance of AOIs in terms of distance normalized indegree, closeness, and betweenness value (as shown by their zero values in the vertical axis in Figure 10).

Figure 11 shows the measures (i.e. indegree, closeness, and betweenness) visualized based on participants for the first time interval. Although there are slight variations, we can see a consistent trend among the participants. In addition, it becomes more evident that AOI ***Q*** and AOI ***a*** can be important AOIs (considering the values of AOI ***(Q;a)***) in addition to AOI ***F***, AOI ***K***, AOI ***b*** and AOI ***d***. In detail, overlapping AOI ***(Q;a)*** received substantial EF duration (as shown by the vertex color in the DNet visualization) although it received moderate number of EFs on it (as shown by the vertex size in the DNet visualization). On the other hand, comparing the three importance measurements, AOI ***(Q;a)***, in spite of having moderate indegree and closeness value, has insignificant betweenness value.

This might be due to the short lifespan of the overlapped AOI state since AOI ***a*** (i.e. SWA2920 flying at 21000 ft with speed 424 knots overtakes AOI ***Q*** (i.e. N46332 flying at 7000 ft with speed 163 knots very quickly on the display. As a result, it is highly unlikely that it will play a crucial role as a bridge for the flow of attention between other groups of aircraft.

## 6. Discussion

Results obtained from the DNet visualization coupled with multiple time interval visualizations enabled us to identify which are the important targets in each of the time intervals and how their importance evolves as we move across time intervals. Application of the three adapted target importance measures (i.e. indegree, closeness, and betweenness) along with the various ways to visualize those values showed that we can better analyze target importance measures that accounts for the interaction among targets.

In addition, we could identify various EM network characteristics of each participant and how the relative importance of various targets differs among them.

DNet was better to visualize the EM flow of the overall network and EF transitions, whereas the adapted dot plot was better to visualize the evolution of importance of each AOI across various time intervals. It is noted that the results could have been different if we had used a different time interval. For example, if the time interval was to 10 minutes, the most important AOI would turn out to be AOI ***d*** and the importance of AOI ***F*** and AOI ***U*** would have been less substantial. However, such results can be misleading since AOI ***F*** and AOI ***U*** were important AOIs during the first 5 minutes.

Selection of time interval thresholds (whether fixed or varied based on events/triggers) can be tricky and depend on the scenario characteristics.

The adapted bar plots were better when multiple target importance measures and/or multiple participants’ measures were needed to be compared side-by-side. The normalized and adapted measure plots show that, despite receiving substantial amount of EFs, an AOI may not be significant in terms of the flow of visual attention across the various AOIs within the display as shown by the closeness and betweenness measures. The proposed approach can be useful in increasing the training efficiency of the novice controllers. Novice controllers can know which are the important targets that needs to be focused upon and how to move the attention across various targets as the scenario characteristics change. Furthermore, the trainees can better understand which targets are highly correlated for conflict mitigation through observing the EF transition characteristics using the DNet and evaluating the closeness/ betweenness values.

## 7. Conclusion

In this research, we integrated the DNet framework with three target important measures (i.e. indegree, closeness, and betweenness). During the integration, we normalized the measurements and then adapted the dot plot and bar plot to better visualize the outputs. The approach facilitated the understanding of how visual attentions occur on the dynamic network (i.e. EM network created from an aircraft conflict task) from various perspectives. The results obtained showed that, the traditional approach of using the raw EM data measures (i.e. number and duration of EFs) might be misleading for dynamic scenarios where the targets’ lifespan on the display are non-uniform. The proposed approach enabled us to better understand how the observers’ attention were devoted to the various targets including the overlapping targets on the display. In addition, in case of dynamic targets, to understand target importance we need to also consider which targets are integral in the smooth flow of attention across the various targets within the display.

## 8. Limitations and Future work

One challenging issue was determining the time intervals for effective analysis. We have used five minute time intervals for the experiment based on trial and errors and we could have applied time intervals based on specific events (e.g. when target appears or disappears, or when a verbal control command is issued by an expert ATCS).

However, event-based time intervals can create issues if too many events occur within very short times or too few events occur in very long times. Instead, we could try applying shorter time intervals. For example, reducing the 5 minute interval to 1 minute interval). However, we might be burdened with visualizing and analyzing too many outputs. Therefore, formal sensitivity analysis needs to be performed as part of future study to determine an optimal range of values for the intervals.

In addition, we have not used pre-determined thresholds to identify important AOIs and only identified them if all measures were relatively higher than others. Therefore, a statistical method should be developed that clearly differentiate the important AOIs from the non-important one.

Moreover, as a future study, we can also analyze the variation of the saccade length and eye fixation duration with time and various air traffic scenario characteristics. For example, we can consider how the distance between several aircraft on the display affects the saccade length of the ATCS. This might help us analyze the type of search behaviors undertaken by them, i.e. is the search goal oriented where they search for targets having similar characteristics or is it a random one.

With increase in the number of AOIs and EF transition between them, the visual scalability of the DNet visualization gets impacted negatively, as there are more instances of edge crossings in the network representations. Furthermore, increase in the number of time intervals also increases the number of static networks within a DNet framework. This leads to increase in the cognitive load of the observers as they have to keep track of an AOI across more number of network representations. Thus, in terms of visualization scalability, we can apply various graph simplification processes, also known as graph filtering processes, where the unimportant edges (i.e. edges representing low EF transitions) are not considered for visualization purpose, thus reducing the visual clutter.

Furthermore, it can be valuable to analyze the community structure of AOIs (cluster of AOIs having high amount of EF transitions between them) and their evolution formed in the network representation of the EF data. Finally, we are planning to develop a mapping scheme between the visual scanning pattern classifications(38) with the DNet results. This might help in understanding how different visual scanning strategies are related to the overall target’s importance

## Ethics and Conflict of Interest

The author(s) declare(s) that the contents of the article are in agreement with the ethics described in http://biblio.unibe.ch/portale/elibrary/BOP/jemr/ethics.html and that there is no conflict of interest regarding the publication of this paper.

## Acknowledgements

This research is supported by the Federal Aviation Administration Center of Excellence (Project No. A17-0162) and the FAA Aviation Research Grants Program (Project No. 15-P-0009). The FAA has sponsored this project through the Center of Excellence for Technical Training and Human Performance. However, the agency neither endorses nor rejects the findings of this research. This information is provided in the interest of invoking technical community comment on the results and conclusions of the research. We worked closely with the FAA Civil Aerospace Medical Institute at Mike Monroney Aeronautical Center (AAM-520).
